# CRM1-Dependent Cytoplasmic Accumulation of TRIM27 Is Associated with Glomerular Endothelial Cell Injury in Lupus Nephritis

**DOI:** 10.3390/cells15181696

**Published:** 2026-09-18

**Authors:** Huixin Cui, Xiaojuan Feng, Yuexin Tian, Xinyan Miao, Lingling Xing, Xiaorong Wang, Yu Tian, Qingjuan Liu, Wei Zhang, Jinxi Liu, Shuxia Liu

**Affiliations:** Key Laboratory of Kidney Diseases of Hebei Province, Department of Pathology, Center of Metabolic Diseases and Cancer Research, Institute of Medical and Health Science, Hebei Medical University, Shijiazhuang 050017, China

**Keywords:** lupus nephritis, glomerular endothelial cells, TRIM27, protein phosphorylation, FOXO1 ubiquitination, AKT signaling

## Abstract

**Highlights:**

**What are the main findings?**

- In GECs, TRIM27 appears to be exported from the nucleus via CRM1 and may contribute to GEC injury in LN through its involvement in FOXO1 ubiquitination.

**What are the implications of the main findings?**

- Targeting the TRIM27 Ser^493^–CRM1 interaction that mediates TRIM27 translocation may alleviate GEC injury in LN, offering new insights into the pathogenesis of LN and potential therapeutic targets.

**Abstract:**

Protein function is closely tied to subcellular localization. Tripartite motif-containing 27 (TRIM27), a member of the tripartite motif family, has been implicated in lupus nephritis (LN), yet the mechanistic basis of its pathogenic role remains incompletely understood. We previously observed that TRIM27 overexpression affects glomerular endothelial cell (GEC) injury and that TRIM27 redistributes from the nucleus to the cytoplasm in LN-affected GECs. Here, using human renal glomerular endothelial cells (HRGECs) and MRL/lpr mice, we show that cytoplasmic TRIM27 may contribute to GEC damage. Mechanistically, TRIM27 appears to translocate to the cytoplasm through association with chromosome region maintenance 1 (CRM1), and this interaction may be promoted by putative phosphorylation at Ser^493^. Suppression of this phosphorylation reduces the TRIM27–CRM1 association and alleviates GEC injury. Furthermore, we found that protein kinase B (AKT) may act upstream of this process and that cytoplasmic TRIM27 may contribute to forkhead box protein O1 (FOXO1) ubiquitination and degradation, which may, in turn, lead to endothelial injury. Collectively, our findings suggest a pathway in which PI3K-dependent phosphorylation of TRIM27 is coupled to CRM1-mediated cytoplasmic accumulation, with AKT likely contributing to this process, and this cascade may lead to FOXO1 degradation and GEC damage in LN. This study provides new insights into the subcellular regulation of TRIM27 and identifies potential intervention points for mitigating glomerular injury.

## 1. Introduction

Lupus nephritis (LN) is a complication of systemic lupus erythematosus that gradually develops into end-stage renal disease and contributes to mortality [1,2,3]. As one of the components of the glomerular filtration barrier, glomerular endothelial cells (GECs) are highly differentiated endothelial cells with fenestrations and a glycocalyx on their surface [4]. Damaged endothelial cells display features such as absence of glycocalyx, disrupted cytoskeleton, and increased permeability, leading to proteinuria [5,6]. Extensive research has revealed that impaired endothelial cells are involved in the development of various kidney diseases [7,8]. Apart from the influence of the systemic autoimmune system, GEC injury contributes to kidney involvement in LN [9]. However, the mechanism by which GECs mediate albuminuria in LN is only partially understood, and further investigation is needed to identify new treatments.

As a member of the tripartite motif protein family, tripartite motif-containing 27 (TRIM27) consists of three conserved domains: a coiled-coil domain, a RING finger, and a B-box zinc finger. It plays a role in a variety of biological processes, such as immune responses, tumorigenesis, cell proliferation, and protein ubiquitination [10,11,12]. Our team previously revealed that TRIM27 overexpression in GECs induced by podocyte-derived exosomes participates in GEC dysfunction in LN [9,13]. TRIM27 was primarily located in the cytoplasm of GECs in the LN group but was found in the nuclei in the control group [13]. Whether cytoplasmic TRIM27 impacts GEC injury and how TRIM27 is transferred from the nucleus to the cytoplasm remain unknown.

In this study, we investigated the role and regulatory mechanism of cytoplasmic TRIM27 in GEC injury in LN. Putative phosphorylation of TRIM27 at Ser^493^ appeared to promote its association with chromosome region maintenance 1 (CRM1), potentially inducing TRIM27 translocation; this may promote forkhead box protein O1 (FOXO1) polyubiquitination, leading to GEC damage in LN. In addition, protein kinase B (AKT) activation may facilitate TRIM27 phosphorylation, providing a new basis for understanding LN pathogenesis and potential therapeutic targets.

## 2. Materials and Methods

### 2.1. Patients

Renal biopsy samples were collected from 30 patients with biopsy-confirmed LN in the Department of Nephrology at the Second Hospital of Hebei Medical University (Shijiazhuang, China) between 2019 and 2023. These patients had received no treatment, infections, or other complications. Ten renal biopsy samples (3 males and 7 females; age range 11–35 years, mean ± SD = 24.6 ± 8.15 years) were obtained from age- and sex-matched patients who underwent nephrectomy for nephrolipoma, nephromyoma, and renal cell carcinoma. Kidney tissue distant from the tumor was confirmed pathologically as normal renal tissue and was used as the control group. Renal sections were used for immunohistochemical staining.

Four of these patients had been part of the cohort in reference [9], with baseline data matching those already published. The other 26 were recruited for the first time and have not been reported elsewhere. All molecular results presented here are new, with no overlap with the experiments in reference [9].

Plasma samples were collected from five patients with active LN in the Department of Immunology and Rheumatology, the Second Hospital of Hebei Medical University (Shijiazhuang, China), between 2019 and 2023. These patients had no infection or other complications and had not been exposed to immunosuppressive therapy. LN activity was assessed using the renal component of the SLE Disease Activity Index (rSLEDAI). The rSLEDAI score is based on four parameters—hematuria (>5 RBCs/hpf), pyuria (>5 WBCs/hpf), proteinuria (>0.5 g/24 h), and urinary casts—with 4 points assigned for each. An rSLEDAI score ≥ 4 signifies active renal disease. The enrolled LN patients were treatment-naive and had no active infections or other complications at initial presentation. Five healthy controls (5 females; age range 27–41 years, mean ± SD = 34.6 ± 5.41 years), age- and sex-matched to the LN patients, were randomly selected. They had no history of autoimmune disease, renal disorders, or long-term medication use. This study was approved by the Ethics Committee of the Second Hospital of Hebei Medical University, and written informed consent was obtained from all participants (approval ID: HebMU 20190027).

The five LN samples were pooled 1:1 (equal volumes), and control plasma was pooled identically. All samples were stored at −80 °C and thawed at 4 °C prior to use. The LN donors did not overlap with the biopsy cohort. Prior to use, all plasma preparations were filtered through a 0.22-μm sterile filter. Cells were exposed to 10% pooled plasma (final concentration) for 48 h. The same plasma pool was used for all experiments, which were repeated three times as independent experimental/culture repeats, each repeat being a separate culture/treatment cycle from freshly thawed aliquots.

### 2.2. Animal Studies

Seven female MRL/MPJ and MRL/lpr mice were purchased from Jackson Laboratory (000485; Bar Harbor, ME, USA). They were housed under specific pathogen-free conditions (22 ± 2 °C, 12-h light/dark cycle) with food and water ad libitum. Sample size was calculated using power analysis (β = 0.8, α = 0.05) with effect sizes estimated from preliminary proteinuria data. All phenotypic and histological evaluations were conducted by two independent investigators blinded to the experimental groups. Animal experiments were approved by the Institutional Animal Care and Use Committee of Hebei Medical University (approval ID: HebMU 2021032). Twenty-four-week-old MRL/MPJ and MRL/lpr mice were sacrificed, and renal tissue was collected for analysis.

### 2.3. Cell Culture and Groups

Human renal glomerular endothelial cells (HRGECs) were purchased from Wuhan Pricella Biotechnology Co., Ltd. HRGECs were cultured in endothelial cell medium (1001; ScienCell; San Diego, CA, USA) containing 5% fetal bovine serum (0025; ScienCell; San Diego, CA, USA), 1% endothelial cell growth factor (1052; ScienCell; San Diego, CA, USA), and 1% penicillin/streptomycin solution (0503; ScienCell; San Diego, CA, USA) in a 37 °C incubator with 5% carbon dioxide.

To verify the effect of LN plasma on TRIM27 localization in HRGECs, cells were stimulated with control or LN plasma. Immunofluorescence (IF) staining and Western blot were used to detect TRIM27 protein expression.

To clarify the effect of TRIM27 translocation on HRGEC dysfunction in LN, cells were divided into four groups: control, LN, LN + leptomycin B (LMB, an inhibitor of nuclear protein export) (L7940; Solarbio; Beijing, China), and LN + absolute ethanol (ET). Cells were pretreated with 5 ng/mL LMB or ET (solvent control) for 12 h, followed by LN plasma treatment. TRIM27 expression was examined by IF and Western blot. Vascular cell adhesion molecule-1 (VCAM-1) and syndecan-1 expression were also assessed using Western blot and IF. Total NO-related metabolites were measured; syndecan-1 and VCAM-1 in the supernatant were detected by enzyme-linked immunosorbent assay (ELISA); and HRGEC permeability was detected by fluorescein isothiocyanate–bovine serum albumin (FITC-BSA).

To investigate the role of CRM1 in TRIM27 translocation, HRGECs were divided into control and LN groups, and co-immunoprecipitation (co-IP) and proximity ligation assay (PLA) were used to detect the interaction between TRIM27 and CRM1. Cells were also divided into four groups: control, LN, LN + selinexor (KPT-330, a selective inhibitor of CRM1) (HY-17536; MedChemExpress; Monmouth Junction, NJ, USA), and LN + dimethyl sulfoxide (DMSO). Cells in the LN + KPT-330 group were pretreated with 3 μM KPT-330 for 12 h and then treated with LN plasma. IF and Western blot were used to detect TRIM27 expression.

To determine whether TRIM27 phosphorylation contributes to its cytoplasmic translocation in LN, cells were divided into control and LN groups, and co-IP was used to detect TRIM27 phosphorylation. Subsequently, HRGECs were divided into five groups: LN + Flag-WT-TRIM27, LN + Flag-MT-TRIM27^T269A^, LN + Flag-MT-TRIM27^S341A^, LN + Flag-MT-TRIM27^S493A^, and LN + Flag-MT-TRIM27^S499A^. IF was used to detect TRIM27 localization. Finally, cells were divided into LN + sh-TRIM27 + Flag-WT-TRIM27, LN + sh-TRIM27 + Flag-MT-TRIM27^S493A^, and LN + sh-TRIM27 + Flag-MT-TRIM27^S493D^ groups; TRIM27 localization was detected by IF, and binding between TRIM27 and CRM1 was detected by co-IP and PLA.

To verify the effect of TRIM27 phosphorylation on GEC injury in LN, HRGECs were divided into LN + sh-TRIM27 + Flag-WT-TRIM27, LN + sh-TRIM27 + Flag-MT-TRIM27^S493A^, and LN + sh-TRIM27 + Flag-MT-TRIM27^S493D^ groups, and cell damage was assessed by IF, ELISA, total NO-related metabolite measurement, and permeability.

To determine whether PI3K/AKT contributes to TRIM27 phosphorylation, cells were divided into control, LN, LN + LY294002 (a PI3K inhibitor that indirectly suppresses AKT) (HY-10108; MedChemExpress; Monmouth Junction, NJ, USA), LN + SC79 (an AKT agonist) (HY-18749; MedChemExpress; Monmouth Junction, NJ, USA), and LN + DM groups. HRGECs in the LN + LY294002 and LN + SC79 groups were pretreated with 25 µM LY294002 or 3 μg/mL SC79, respectively, for 1 h. Following this, TRIM27 phosphorylation was detected by co-IP, and its localization was assessed by IF. To confirm that PI3K/AKT-induced TRIM27 translocation was dependent on CRM1, HRGECs were divided into DM, SC79, KPT-330, and SC79 + KPT-330 groups, and TRIM27 expression was detected by IF and Western blot.

To investigate whether cytoplasmic TRIM27 contributes to GEC damage through its involvement in FOXO1 ubiquitination, cells were divided into control and LN groups, and binding between TRIM27 and FOXO1 was detected by co-IP and PLA. Cells were then divided into control, LN, LN + carbobenzoxy-L-leucyl-L-leucyl-L-leucinal (MG132, a proteasome inhibitor) (HY-13259; MedChemExpress; Monmouth Junction, NJ, USA), and LN + DM groups. HRGECs in the LN + MG132 group were pretreated with 100 nM MG132 for 12 h, and FOXO1 protein expression was detected by Western blot. Subsequently, cells were divided into control + MG132 and LN + MG132 groups, and FOXO1 ubiquitination was tested by co-IP. Cells were then divided into LN + sh-TRIM27 + cycloheximide (CHX, a protein synthesis inhibitor) (HY-12320; MedChemExpress; Monmouth Junction, NJ, USA) + Flag-WT-TRIM27 and LN + sh-TRIM27 + CHX + Flag-MT-TRIM27^S493A^ groups. Each group was treated with 3 μg/mL CHX for 0, 12, 24, and 48 h, and FOXO1 expression was detected by Western blot. Finally, HRGECs were divided into LN + sh-TRIM27 + MG132 + Flag-WT-TRIM27, LN + sh-TRIM27 + MG132 + Flag-MT-TRIM27^S493A^, and LN + sh-TRIM27 + MG132 + Flag-MT-TRIM27^S493D^ groups, and FOXO1 ubiquitination was detected by co-IP.

### 2.4. Immunohistochemical Staining

Paraffin-embedded kidney tissue sections fixed with 4% paraformaldehyde were deparaffinized with xylene and rehydrated with a graded ethanol series. Antigen retrieval was then performed with a pressure cooker, and endogenous peroxidase was blocked by incubation with 3% H_2_O_2_ for 30 min at 37 °C. Sections were then blocked with 10% goat serum and incubated with anti-TRIM27 (1:100; 12205-1-AP; Proteintech; Wuhan, China) and anti-CD31 (1:500; ab281583; Abcam; Cambridge, UK) antibodies overnight at 4 °C. Sections were then incubated at 37 °C with horseradish peroxidase-conjugated anti-rabbit/mouse immunoglobulin G (IgG) and stained with diaminobenzidine. Images were captured using an Olympus microscope (Olympus; Tokyo, Japan).

### 2.5. IF Staining

Frozen sections were blocked with 10% goat serum at 37 °C for 1 h and then incubated with anti-TRIM27 (1:100; 12205-1-AP; Proteintech; Wuhan, China) and anti-CD31 (1:100; 66065-2-Ig; Proteintech; Wuhan, China) antibodies at 4 °C overnight. HRGECs were cultured on enzyme-sterilized slides in six-well plates, then fixed with 4% paraformaldehyde for 20 min at room temperature, followed by permeabilization with 0.3% Triton X-100 for 15 min at room temperature. Next, 10% goat serum was added for blocking at 37 °C for 1 h, followed by incubation with anti-TRIM27 (1:100; 12205-1-AP; Proteintech; Wuhan, China), anti-syndecan-1 (1:200; ab128936; Abcam; Cambridge, UK), anti-Flag (1:200; T0003; Affinity; Liyang, China), and anti-ZO-1 (1:200; 21773-1-AP; Proteintech; Wuhan, China) antibodies overnight at 4 °C.

The next day, FITC-goat anti-rabbit IgG and TRITC-goat anti-mouse IgG were added to frozen sections or cells at 37 °C for 2 h. Finally, DAPI was added and incubated at 4 °C for 30 min. Sections were observed under a microscope (Olympus; Tokyo, Japan).

### 2.6. Separation of Nuclear and Cytoplasmic Proteins

HRGECs were collected into a centrifuge tube with a cell scraper, and 200 μL of cytoplasmic protein extraction Reagent A (P0028; Beyotime Biotechnology; Shanghai, China) was added per 20 μL of cells. Cells were then thoroughly lysed on ice for 15 min before 10 μL of cytoplasmic protein extraction Reagent B (P0028; Beyotime Biotechnology; Shanghai, China) was added, and the mixture was centrifuged at 4 °C and 12,000× *g* for 20 min. The supernatant was collected into a new centrifuge tube, which contained cytoplasmic proteins. Subsequently, 50 μL of nuclear extraction reagent was added to the pellet, which was fully lysed in an ice bath for 30 min before the lysate was centrifuged at 4 °C and 12,000× *g* for 10 min. The supernatant was aspirated into a centrifuge tube; this fraction contained nuclear proteins. Cytoplasmic and nuclear proteins were used for Western blot analysis.

### 2.7. Western Blot

Denatured protein samples were separated by 10% or 12% sodium dodecyl sulfate–polyacrylamide gel electrophoresis and transferred to polyvinylidene difluoride membranes. Membranes containing protein samples were blocked with 5% BSA or 5% skim milk at 37 °C for 1.5 h and then incubated with anti-TRIM27 (1:1000; 12205-1-AP; Proteintech; Wuhan, China), anti-GAPDH (1:10,000; 10494-1-AP; Proteintech; Wuhan, China), anti-histone H3 (1:1000; 17168-1-AP; Proteintech; Wuhan, China), anti-VCAM-1 (1:1000; ab134047; Abcam; Cambridge, UK), anti-CRM1 (1:1000; 66763-1-Ig; Proteintech; Wuhan, China), anti-phosphoserine/threonine (1:1000; PP2551; ECM Biosciences; Versailles, KY, USA), anti-phospho-AKT (Ser473) (1:1000; ab81283; Abcam; Cambridge, UK), anti-phospho-AKT (Thr308) (1:1000; 13038; Cell Signaling; Boston, MA, USA), anti-AKT1 (1:1000; 60203-2-Ig; Abcam; Cambridge, UK), anti-Flag (1:1000; T0003; Affinity; Liyang, China), anti-FOXO1 (1:1000; 2880; Cell Signaling; Boston, MA, USA), or anti-ubiquitin (1:1000; sc-271289; Santa Cruz; Dallas, TX, USA) antibodies overnight at 4 °C. Membranes were then incubated with horseradish peroxidase-conjugated goat anti-rabbit or mouse IgG antibodies (1:5000; Proteintech; Wuhan, China) for 1 h at 37 °C. Finally, images were captured using an ECL system (BL520B; Biosharp; Beijing, China).

### 2.8. NO Measurement

HRGECs were thoroughly lysed with NO lysis buffer on ice for 40 min, followed by centrifugation at 4 °C at 12,000× *g* for 20 min. The supernatant was collected, and total NO-related metabolites (nitrite and nitrate) were measured using the Total Nitric Oxide Detection Kit (S0024; Beyotime Biotechnology; Shanghai, China) according to the manufacturer’s instructions. This assay reflects the total pool of NO metabolites rather than bioavailable endothelial NO and does not distinguish between eNOS- and iNOS-derived NO.

### 2.9. ELISA

Syndecan-1 and VCAM-1 in the medium of HRGECs were measured with ELISA kits (ZC-54317, ZC-35268; ZCIBIO Technology; Shanghai, China). The absorbance at 450 nm was measured with a spectrophotometer.

### 2.10. HRGEC Permeability

HRGEC permeability was determined using FITC-BSA (SF063; Solarbio; Beijing, China). HRGECs were seeded in the upper transwell chamber. Once the cells formed a monolayer, 5 mg/mL FITC-BSA was added to the upper chamber, and equimolar label-free BSA was added to the lower chamber. The chamber was then incubated in the dark for 3 h. The upper and lower chamber media were collected and measured using a microplate fluorometer (Berthold; Bad Wildbad, Germany) with filters set to 495 (excitation) and 520 nm (emission). The permeability coefficient (Pa) was used to represent the permeability of HRGEC, calculated as Pa = [A]V/A[L]—where [A] represents the fluorescence intensity of FITC-BSA in the lower chamber, V is the volume of the solution in the lower chamber, A is the floor area of the upper chamber, and [L] represents the fluorescence intensity of the upper chamber. Permeability was calculated as P (%) = (Pa of the experimental group/Pa of the control group) × 100%.

### 2.11. Co-IP

HRGECs were lysed with 1 mL of RIPA buffer. Anti-CRM1, anti-TRIM27, anti-phosphoserine/threonine, anti-FOXO1, anti-ubiquitin, or anti-IgG antibodies were added to the lysate (700 μL) and incubated at 4 °C overnight on a shaker. The next day, protein A/G beads (sc-2003; Santa Cruz; Dallas, TX, USA) were added to the lysate and incubated at 4 °C for 4 h. Subsequently, the lysate was washed with washing buffer, and the protein pellet was collected after centrifugation at 500× *g* for 20 min. Proteins were denatured at 100 °C for subsequent Western blot analysis.

### 2.12. PLA

HRGECs were cultured on enzyme-sterilized slides in 24-well plates. Cells were fixed with 4% paraformaldehyde for 15 min at room temperature and then permeabilized with 0.3% Triton X-100 for 15 min. Duolink^®^ PLA reagent (DUO92002; Merck KGaA; Darmstadt, Germany) was then applied following the manufacturer’s instructions. The sections were observed by microscopy (Olympus; Tokyo, Japan).

### 2.13. Plasmids and Transfection

The Flag-WT-TRIM27 plasmid was purchased from Fulengen Co. (Guangzhou, China). The Flag-MT-TRIM27 plasmid was purchased from Youbio Biotech (Changsha, China). The sh-TRIM27 plasmid was purchased from Cyagen Biotechnology (Suzhou, China). HRGECs were transfected with the above plasmids using Lipofectamine 3000 (L3000015; Invitrogen; Carlsbad, CA, USA) according to the manufacturer’s protocol.

### 2.14. Statistical Analysis

Statistical analysis was performed using SPSS 21.0 (SPSS, Inc., Chicago, IL, USA). Quantitative data are presented as mean ± standard deviation (SD). Normality and homogeneity of variances were assessed using the Shapiro–Wilk and Levene tests, respectively, to justify parametric procedures. For normally distributed and homoscedastic data, two-group comparisons used Student’s *t*-test, while multi-group comparisons employed one-way analysis of variance (ANOVA) with Tukey’s honestly significant difference (HSD) post hoc test when the overall *F*-test was significant. Two-way ANOVA (including interaction) was used for designs with two categorical factors. Pearson’s correlation was used to examine linear associations between continuous variables. All tests were two-tailed, with significance set at *p* < 0.05.

## 3. Results

### 3.1. TRIM27 Translocation May Contribute to GEC Injury in LN In Vitro

**Figure 1:**
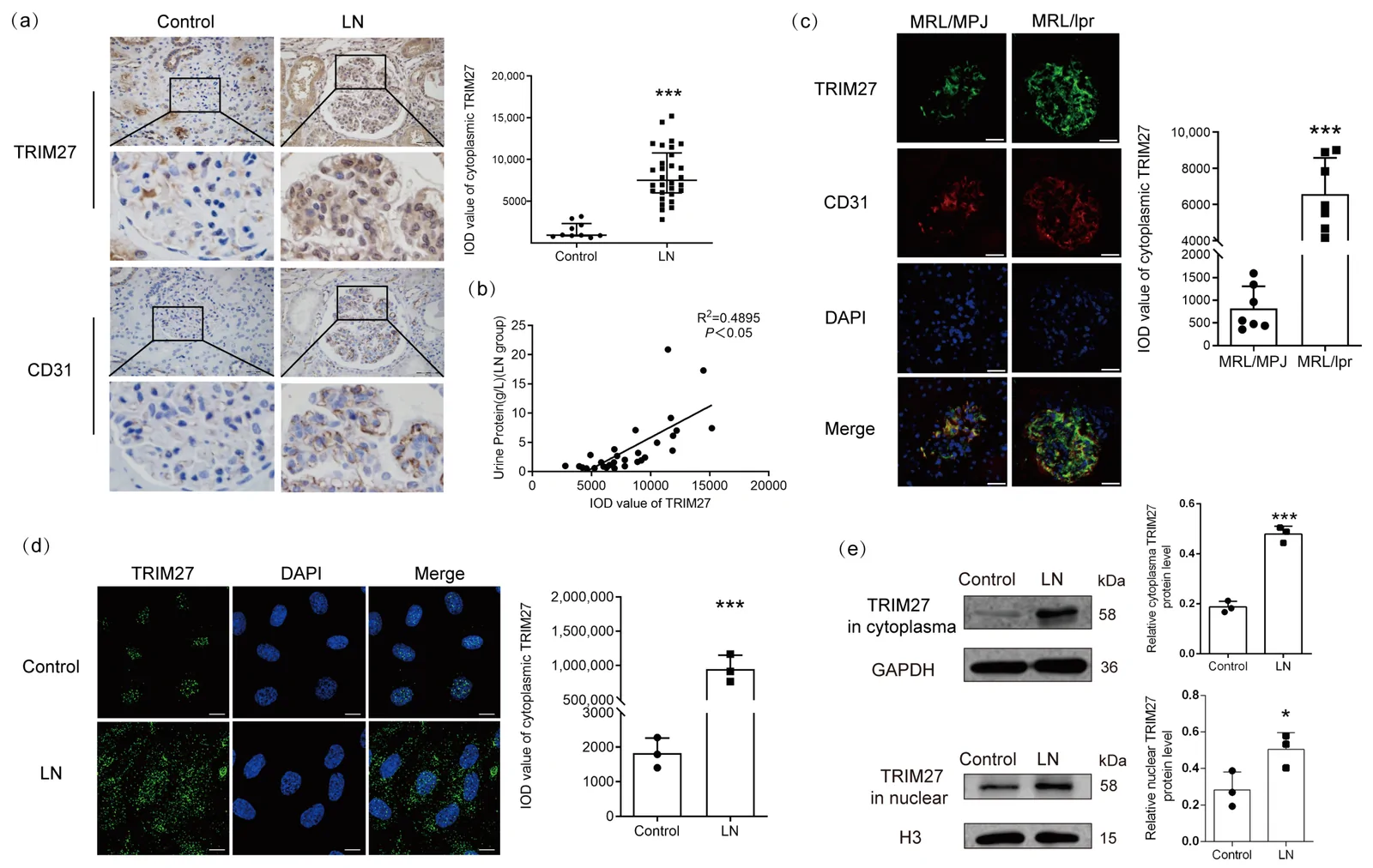
TRIM27 was translocated from the nucleus to the cytoplasm in glomerular endothelial cells in LN. (**a**) Immunohistochemical (IHC) staining showing that cytoplasmic tripartite motif-containing 27 (TRIM27) in glomerular endothelial cells was increased in patients with lupus nephritis (LN) compared with the control group. Scale bars: 50 μm. For each biopsy sample, at least five randomly selected fields were examined, and at least one glomerulus was analyzed per field. Values are median (IQR). *** *p* < 0.001 vs. control group; Mann–Whitney U test. (**b**) Positive correlation between TRIM27 expression in glomerular endothelial cells and urine protein in patients with LN. Pearson’s correlation was used to examine linear associations between continuous variables. *p* = 0.002. (**c**) Immunofluorescence (IF) results demonstrating that cytoplasmic TRIM27 (green) was increased in glomerular endothelial cells (red) in MRL/lpr mice compared with MRL/MPJ mice. Nuclei were stained with DAPI (blue).Scale bars: 25 μm. For each tissue sample, at least ten randomly selected fields were examined, and a minimum of three glomeruli were analyzed per field. Values are mean ± SD. *** *p* < 0.001 vs. MRL/MPJ group; Student’s *t*-test. (**d**,**e**) TRIM27 expression in the cytoplasm was increased in human renal glomerular endothelial cells (HRGECs), as detected by IF and Western blot. Data were obtained from three independent experimental/culture repeats using the same pooled plasma preparations, with at least 10 randomly selected fields and 5 cells per field analyzed for each condition. Scale bars: 10 μm. The data are presented as the mean ± SD. * *p* = 0.045, *** *p* < 0.001 vs. control group; Student’s *t*-test. For (**a**,**b**), control group: *n* = 10; LN group: *n* = 30. For (**c**), *n* = 7. For (**d**,**e**), *n* = 3. Experiments/cultures were repeated three times independently. Each point represents an individual sample.

Immunohistochemistry was employed to verify TRIM27 expression and its localization in GECs from patients with LN. TRIM27 was primarily localized in the nucleus of GECs in the control group but was increasingly found in the cytoplasm of GECs from patients with LN (Figure 1a). TRIM27 expression in GECs was positively correlated with urinary protein/24 h in patients with LN (Figure 1b). Figure 1c shows that cytoplasmic TRIM27 in GECs was dramatically increased in MRL/lpr mice compared with MRL/MPJ mice. IF and Western blot revealed that LN plasma could induce TRIM27 cytosolic accumulation in HRGECs (Figure 1d,e).

**Figure 2:**
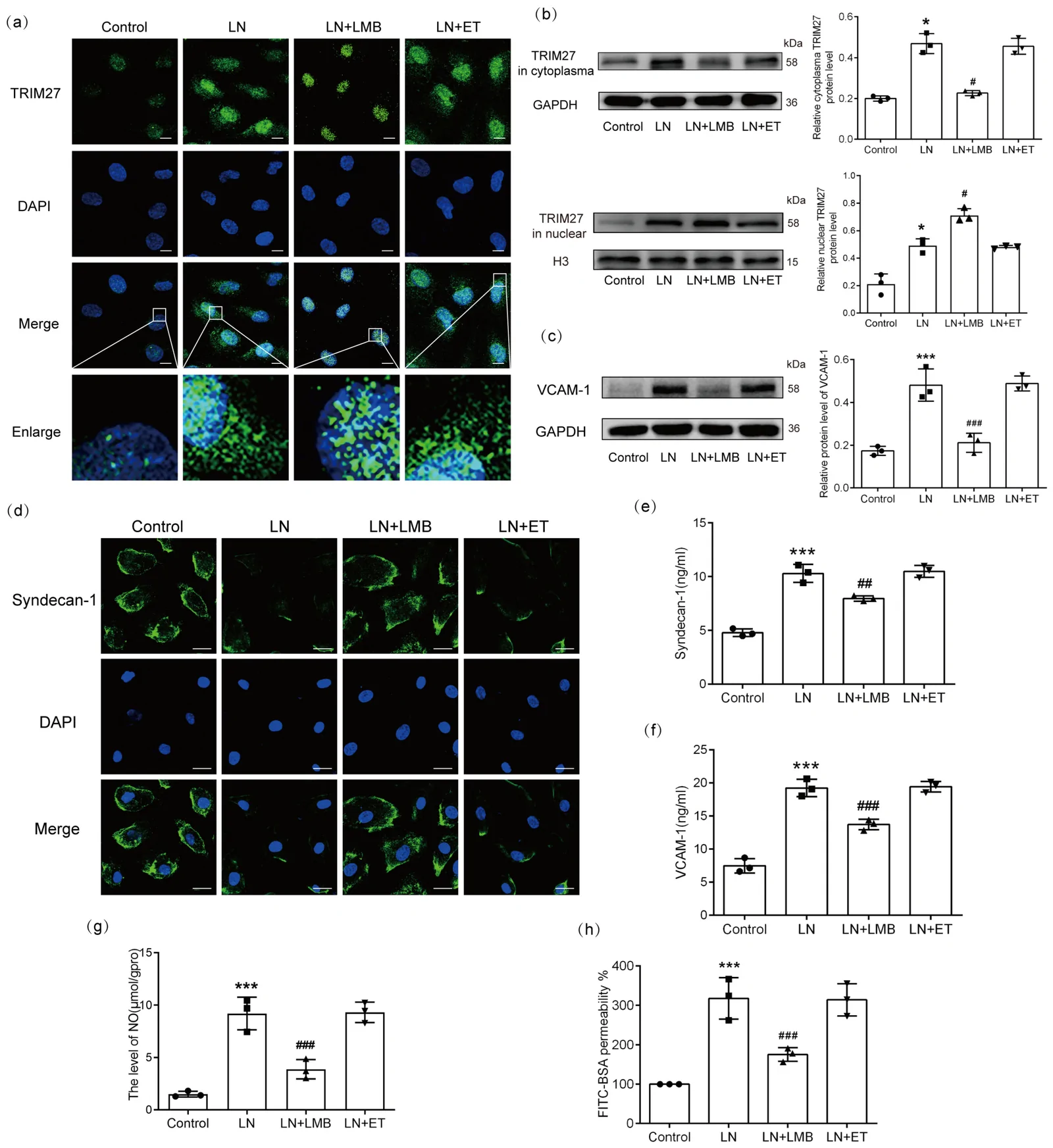
TRIM27 translocation may contribute to endothelial cell damage in LN in vitro. (**a**,**b**) IF and Western blot results showing that leptomycin B (LMB) effectively inhibited TRIM27 translocation from the nucleus to the cytoplasm in HRGECs treated with LN plasma(* *p* = 0.033 vs. control group, # *p* = 0.023 vs. LN + absolute ethanol (ET) group; scale bars: 10 μm). Green, TRIM27; blue, nuclei; boxed area, higher magnification view. (**c**) Vascular cell adhesion molecule-1 (VCAM-1) expression was increased in HRGECs treated with LN plasma and was reduced after inhibition of TRIM27 translocation by LMB. (**d**) Syndecan-1 expression was reduced in HRGECs stimulated with LN plasma, which was increased after suppression of TRIM27 translocation by LMB (scale bars: 25 μm). Green, Syndecan-1; blue, nuclei. (**e**,**f**) Enzyme-linked immunosorbent assay (ELISA) results revealing syndecan-1 and VCAM-1 levels in the supernatant of HRGECs increased in the LN group and decreased in the LN + LMB group (## *p* = 0.005 vs. LN + absolute ethanol (ET) group.). (**g**,**h**) LN plasma stimulation induced increased HRGEC nitric oxide (NO) content and permeability, which was abolished by LMB pretreatment. The data are presented as the mean ± SD; one-way ANOVA with Tukey’s post hoc test. For (**b**,**c**,**e**–**h**), *n* = 3. Experiments/cultures were repeated three times independently. *** *p* < 0.001 vs. control group; ### *p* < 0.001 vs. LN + absolute ethanol (ET) group. Each point represents an individual sample.

We further explored whether inhibiting TRIM27 cytoplasmic accumulation influenced HRGEC dysfunction in vitro. IF and Western blot showed that LMB pretreatment suppressed TRIM27 translocation from the nucleus to the cytoplasm (Figure 2a,b). Compared with the control group, VCAM-1 expression was increased in the LN group; LMB pretreatment abolished this upregulation (Figure 2c). Similarly, IF staining revealed that syndecan-1 was mainly located in the cytoplasm and membrane in the control group; LN stimulation reduced its expression, whereas LMB promoted it (Figure 2d). ELISA indicated that syndecan-1 and VCAM-1 were increased in the LN group compared with the control group; LMB application reduced syndecan-1 and VCAM-1 in the supernatant of HRGECs treated with LN plasma (Figure 2e,f). The levels of total NO-related metabolites and cell permeability were increased in HRGECs treated with LN plasma; this effect was suppressed by LMB (Figure 2g,h). Overall, suppression of TRIM27 translocation by LMB pretreatment alleviated HRGEC damage induced by LN plasma, suggesting that cytoplasmic TRIM27 may contribute to HRGEC injury in LN.

### 3.2. TRIM27 Cytoplasmic Accumulation in GECs Was CRM1-Dependent in LN

**Figure 3:**
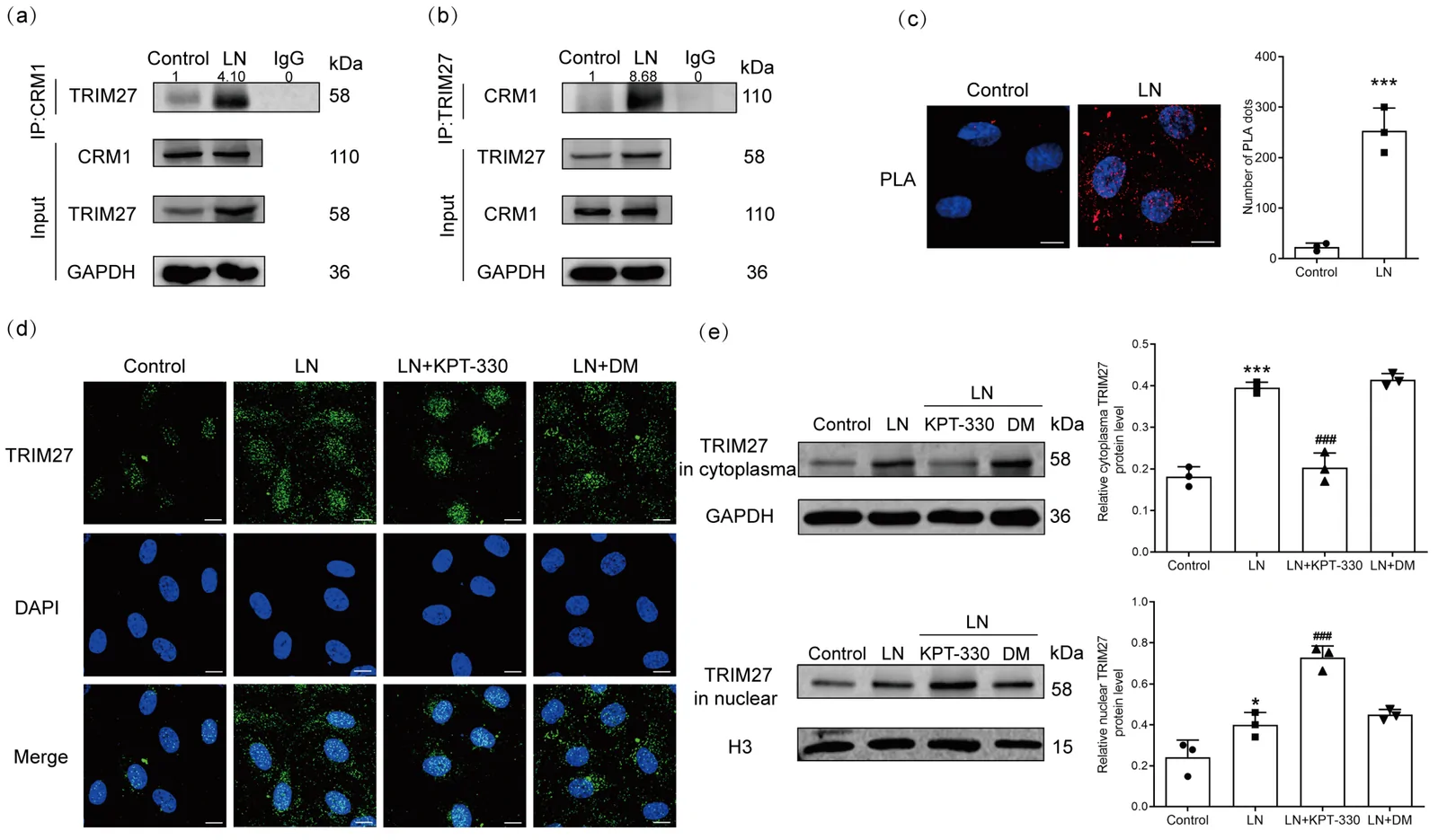
CRM1-mediated TRIM27 cytoplasmic translocation in HRGECs in LN. (**a**–**c**) Co-immunoprecipitation (Co-IP) and proximity ligation assay (PLA) revealing that the interaction between TRIM27 and chromosome region maintenance 1 (CRM1) was increased in HRGECs treated with LN plasma (scale bars: 10 μm). Red, PLA-positive signals; blue, nuclei. Data were obtained from three independent experimental/culture repeats using the same pooled plasma preparations, with at least 10 randomly selected fields and 5 cells per field analyzed for each condition. Student’s *t*-test. (**d**,**e**) IF and Western blot results demonstrating that cytoplasmic TRIM27 in HRGECs was reduced after treatment with KPT-330, a CRM1 inhibitor(scale bars: 10 μm). Green, TRIM27; blue, nuclei. The data are presented as the mean ± SD; one-way ANOVA with Tukey’s post hoc test. For (**e**), *n* = 3. Experiments/cultures were repeated three times independently. * *p* = 0.048, *** *p* < 0.001 vs. control group, ### *p* < 0.001 vs. LN + DM group. Each point represents an individual sample.

CRM1 is an important receptor that mediates nuclear protein export [14]. To investigate the role of CRM1 in TRIM27 cytoplasmic redistribution, co-IP was employed to test whether the two proteins interacted. The interaction between TRIM27 and CRM1 was increased after treatment with LN plasma (Figure 3a,b). PLA revealed that LN plasma induced more red foci, demonstrating their association in the cytoplasm of HRGECs (Figure 3c). To verify the role of CRM1 in TRIM27 translocation, we used KPT-330, a selective CRM1 inhibitor. IF and Western blot results demonstrated that nuclear and cytoplasmic TRIM27 levels in the LN group were increased compared with the control group, whereas KPT-330 significantly suppressed cytoplasmic accumulation of TRIM27 (Figure 3d,e).

### 3.3. Putative Phosphorylation at TRIM27 Ser493 May Contribute to Translocation and Play a Crucial Role in GEC Dysfunction in LN In Vitro

**Figure 4:**
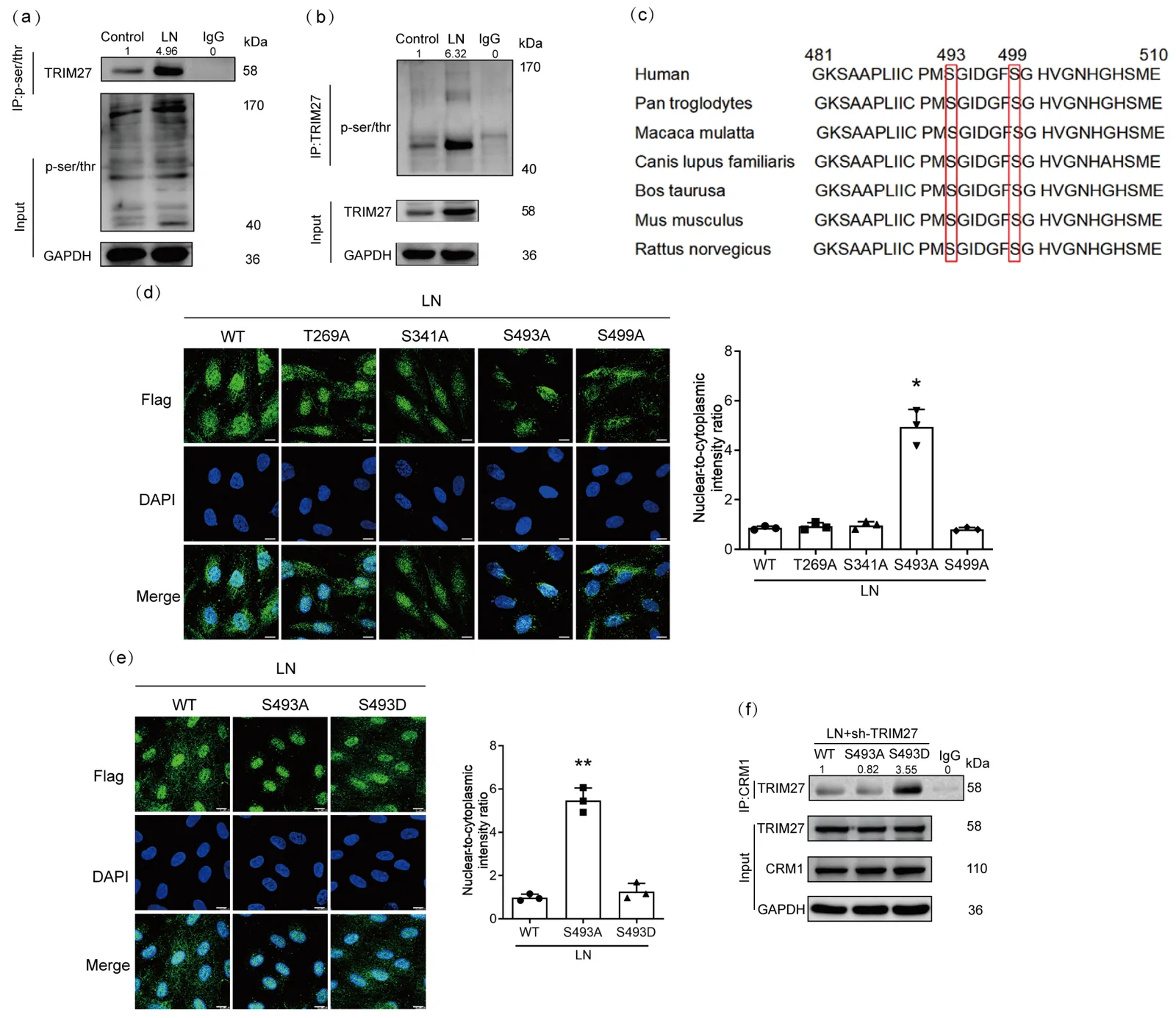
TRIM27 phosphorylation may contribute to its cytoplasmic translocation in HRGECs in vitro. (**a**,**b**) Co-IP results showing that TRIM27 phosphorylation was increased in HRGECs stimulated with LN plasma. (**c**) TRIM27 phosphorylation sites were highly conserved across multiple species. Red boxes, conserved Ser493 and Ser499 residues of TRIM27 across species. (**d**,**e**) IF results showing that transfection with non-phosphorylated MT-TRIM27^S493A^ reduced cytoplasmic TRIM27, whereas transfection with phospho-mimetic MT-TRIM27^S493D^ appeared to promote TRIM27 cytoplasmic accumulation. Data were obtained from three independent experimental/culture repeats using the same pooled plasma preparations, with at least 10 randomly selected fields and 5 cells per field analyzed for each condition. Data are presented as the mean ± SD; one-way ANOVA with Tukey’s post hoc test. * *p* = 0.029 vs. LN + WT group; ** *p* = 0.002 vs. LN + S493D group. Scale bars: 10 μm. Green, Flag; blue, nuclei. (**f**) Co-IP results revealing that the interaction between TRIM27 and CRM1 was decreased in HRGECs transfected with non-phosphorylated MT-TRIM27^S493A^ and appeared to be increased after transfection with the phospho-mimetic MT-TRIM27^S493D^. Each point represents an individual sample.

Protein phosphorylation can mediate changes in localization [15]. We tested whether TRIM27 phosphorylation could lead to its cytosolic accumulation in response to LN plasma. TRIM27 phosphorylation at serine and threonine sites was greatly increased in HRGECs stimulated with LN plasma (Figure 4a,b). We predicted potential phosphorylation sites (www.phosphosite.org) that are highly conserved in mammals (Figure 4c). These sites, including Thr^269^, Ser^341^, Ser^493^, and Ser^499^, were then replaced with alanine, which cannot be phosphorylated. The mutants were transfected into HRGECs to observe TRIM27 localization.

LN plasma triggered cytosolic TRIM27 accumulation in HRGECs transfected with WT-TRIM27, MT-Thr^269A^-TRIM27, MT-Ser^341A^-TRIM27, and MT-Ser^499A^-TRIM27 (Figure 4d). LN plasma caused nuclear TRIM27 accumulation in the MT-Ser^493A^-TRIM27 group, indicating that phosphorylation of TRIM27 at Ser^493^ may contribute to its translocation. Ser^493^ was mutated to phospho-mimetic aspartate, which formed foci in the cytoplasm (Figure 4e). Additionally, co-IP showed that the interaction between TRIM27 and CRM1 was decreased in the MT-Ser^493A^-TRIM27 group but increased in the MT-Ser^493D^-TRIM27 group compared with the WT-TRIM27 group, further suggesting that TRIM27 phosphorylation at Ser^493^ may promote its binding to CRM1 and subsequent translocation (Figure 4f).

To further explore the potential role of TRIM27 phosphorylation at Ser493, HRGECs were transfected with sh-TRIM27-LV to knock down endogenous TRIM27 expression; they were then transfected with WT-TRIM27 vector, non-phosphorylated MT-TRIM27^S493A^, or phospho-mimetic MT-TRIM27^S493D^, followed by stimulation with LN plasma before damage indicators were assessed.

**Figure 5:**
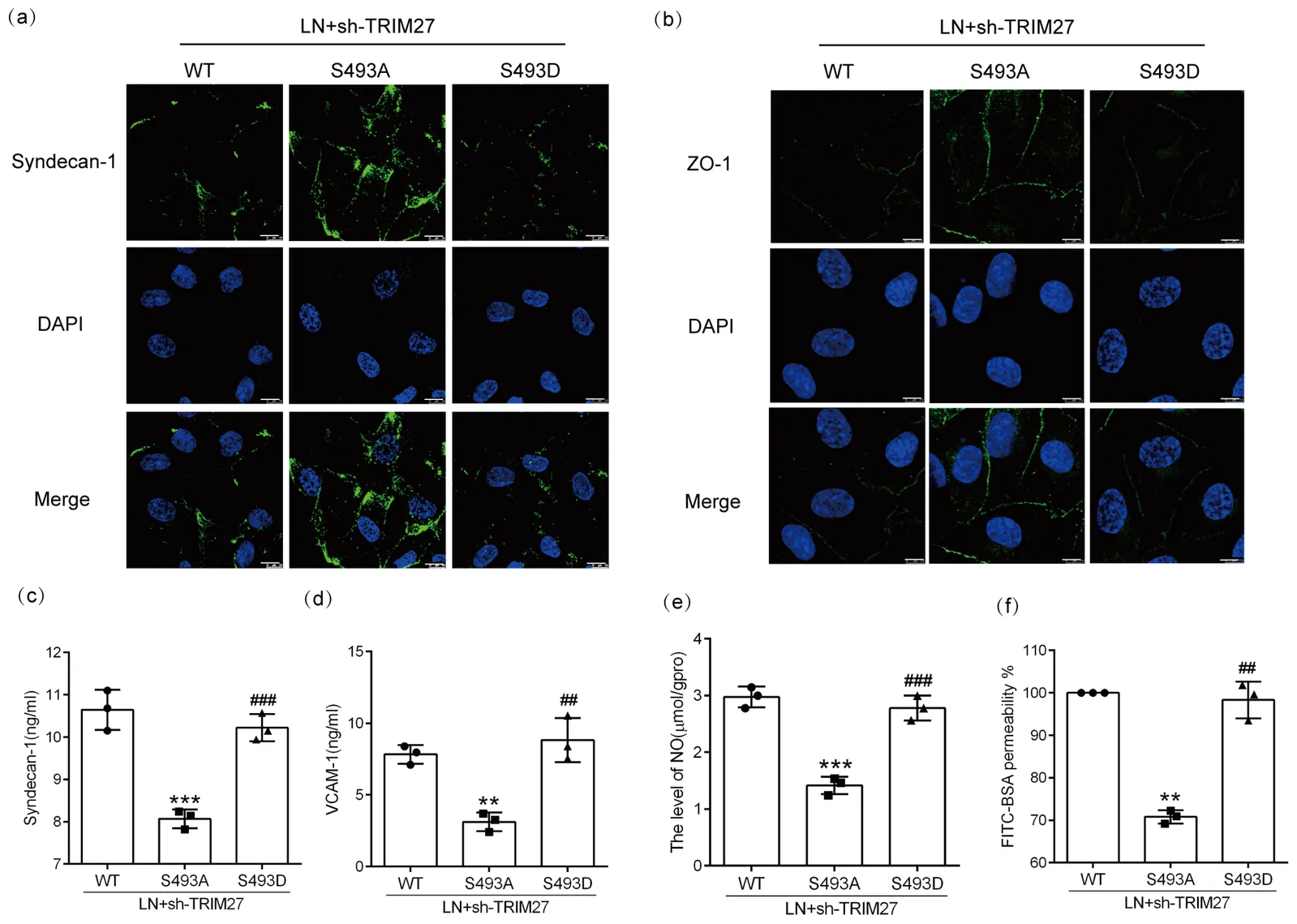
Phosphorylated TRIM27 may be involved in HRGEC injury in vitro. (**a**,**b**) IF results showing that the expression of syndecan-1 (green) and zonula occludens-1 (ZO-1, green) was increased in HRGECs transfected with non-phosphorylated MT-TRIM27^S493A^; these effects were reversed after transfection with the phospho-mimetic MT-TRIM27^S493D^ (scale bars: 10 and 7.5 μm). Blue, nuclei. (**c**,**d**) ELISA results demonstrating that syndecan-1 and VCAM-1 levels in the supernatants of HRGECs were reduced after transfection with non-phosphorylated MT-TRIM27^S493A^ but were increased after transfection with the phospho-mimetic MT-TRIM27^S493D^ (** *p* = 0.002 vs. LN + sh-TRIM27 + Flag-WT-TRIM27 group, ## *p* = 0.005 vs. LN + sh-TRIM27 + Flag-MT-TRIM27^S493A^ group). (**e**,**f**) Total NO-related metabolites and permeability were suppressed in HRGECs transfected with non-phosphorylated MT-TRIM27^S493A^ compared with the WT-TRIM27 group but were increased after transfection with the phospho-mimetic MT-TRIM27^S493D^ (** *p* = 0.003 vs. LN + sh-TRIM27 + Flag-WT-TRIM27 group, ## *p* = 0.009 vs. LN + sh-TRIM27 + Flag-MT-TRIM27^S493A^ group). Data are presented as the mean ± SD; one-way ANOVA with Tukey’s post hoc test. For (**c**–**f**), *n* = 3. Experiments/cultures were repeated three times independently. *** *p* < 0.001 vs. LN + sh-TRIM27 + Flag-WT-TRIM27 group, ### *p* < 0.001 vs. LN + sh-TRIM27 + Flag-MT-TRIM27^S493A^ group. Each point represents an individual sample.

The positive signals for syndecan-1 and ZO-1 were increased in the MT-TRIM27^S493A^ group but reduced in the MT-TRIM27^S493D^ group compared with the WT-TRIM27 group (Figure 5a,b). Syndecan-1 and VCAM-1 levels in the supernatant, total NO-related metabolite level, and HRGEC permeability in the MT-TRIM27^S493A^ group were reduced compared with those in the WT-TRIM27 group but were increased in the MT-TRIM27^S493D^ group (Figure 5c–f). Overall, TRIM27 phosphorylation at Ser^493^ was associated with GEC dysfunction.

### 3.4. Activation of PI3K/AKT Signaling May Induce TRIM27 S493 Putative Phosphorylation In Vitro

**Figure 6:**
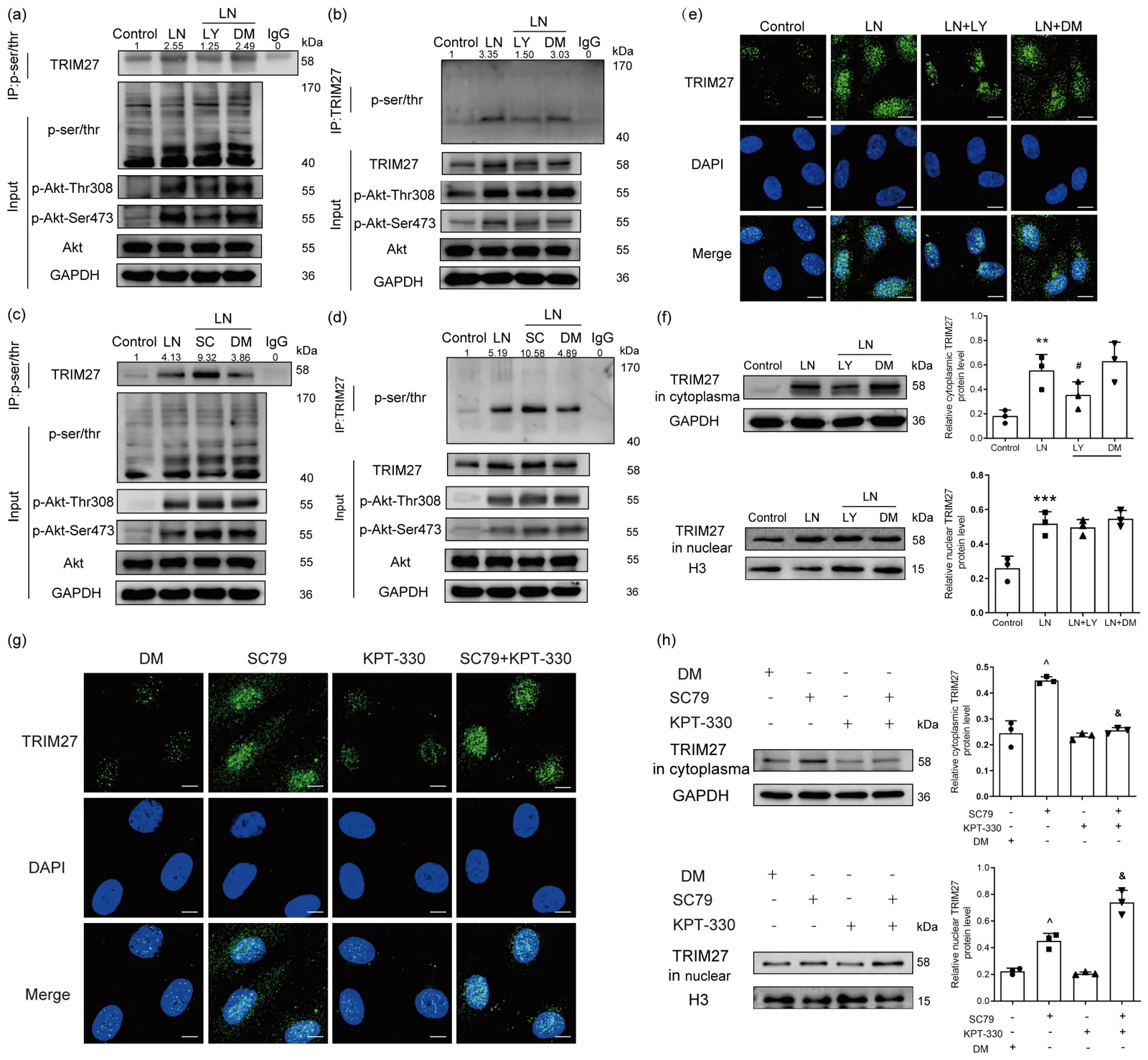
PI3K pathway-dependent TRIM27 phosphorylation with possible AKT involvement in HRGECs in vitro. (**a**–**d**) Co-IP results showing that protein kinase B (AKT) inhibition and activation reduced and increased TRIM27 phosphorylation in HRGECs, respectively. (**e**,**f**) IF and Western blot results revealed that cytoplasmic TRIM27 was decreased in HRGECs after pretreatment with LY294002 (LY) (a PI3K inhibitor that indirectly suppresses AKT) (** *p* = 0.006 vs. control group; # *p* = 0.024 vs. LN + DM group; scale bars: 10 μm). Green, TRIM27; blue, nuclei. (**g**,**h**) TRIM27 accumulation in the cytoplasm was increased in HRGECs stimulated with SC79 (SC) (an AKT agonist), which was suppressed after combination with KPT-330 (a CRM1 inhibitor) (^ *p* = 0.034 vs. DM group and & *p* = 0.048 vs. SC79 group for cytoplasm; ^ *p* = 0.031 vs. DM group and & *p* = 0.023 vs. SC79 group for nucleus; scale bars: 10 μm). Green, TRIM27; blue, nuclei. +, with treatment; −, without treatment. Data are presented as the mean ± SD; one-way ANOVA with Tukey’s post hoc test. For (**f**,**h**), *n* = 3. Experiments/cultures were repeated three times independently. *** *p* < 0.001 vs. control group Each point represents an individual sample.

PI3K/AKT mediates the phosphorylation of various proteins [16]. To determine the regulatory mechanism underlying TRIM27 phosphorylation, a PI3K inhibitor (LY294002) and an AKT agonist (SC79) were used. TRIM27 phosphorylation was decreased and increased by LY294002 and SC79, respectively (Figure 6a–d). Additionally, LY294002 reduced cytoplasmic TRIM27 and enhanced its nuclear expression (Figure 6e,f).

To further verify whether PI3K/AKT may contribute to phosphorylation of TRIM27 at Ser^493^ and its binding to CRM1, leading to its translocation, SC79 and KPT-330 were used in combination to treat HRGECs. SC79 alone promoted the accumulation of cytoplasmic TRIM27, whereas its combination with KPT-330 decreased TRIM27 cytoplasmic accumulation (Figure 6g,h).

### 3.5. Cytoplasmic TRIM27 May Contribute to FOXO1 Ubiquitination In Vitro

**Figure 7:**
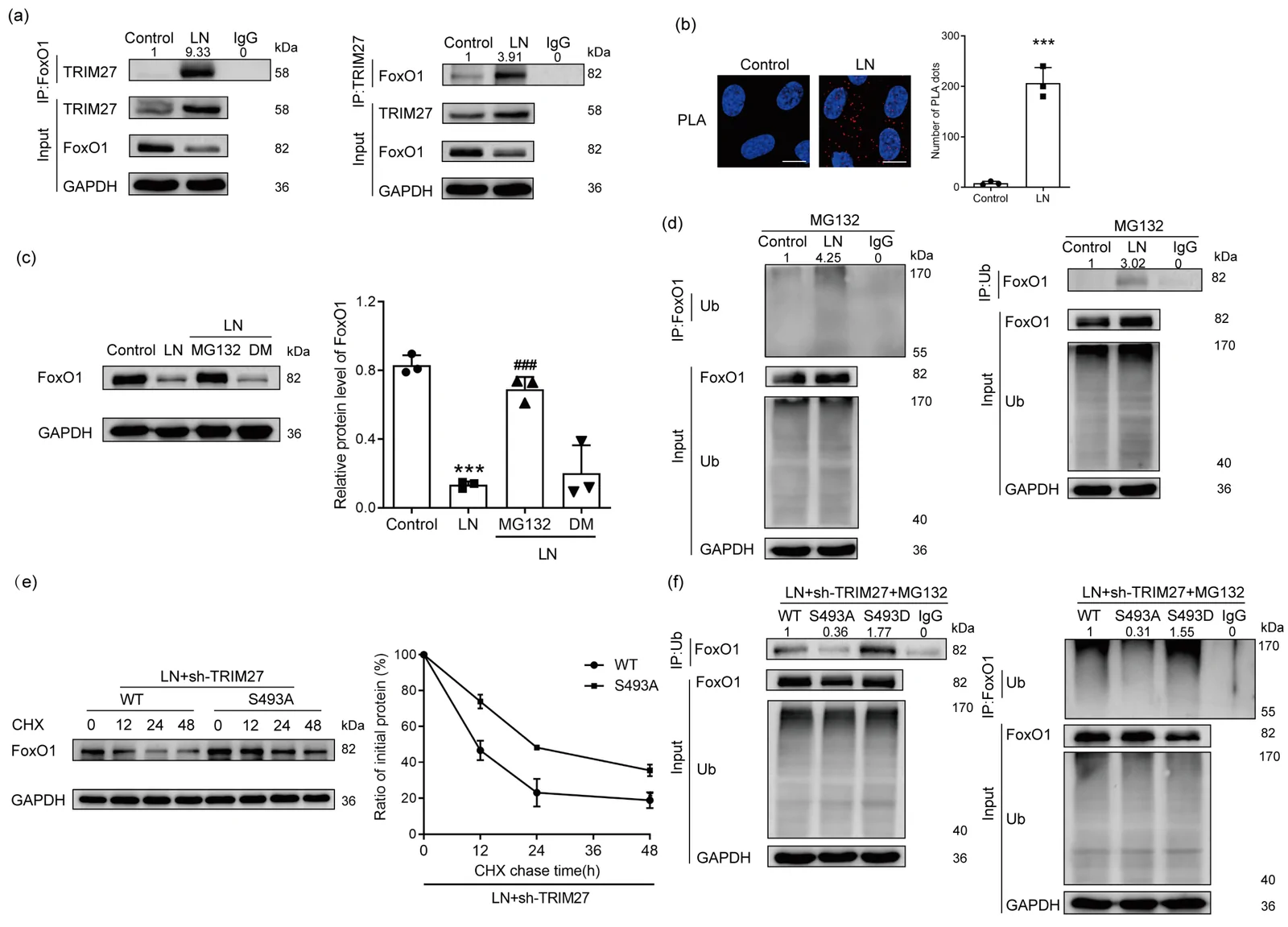
Cytoplasmic TRIM27 may be involved in endothelial cell injury by contributing to FOXO1 ubiquitination in vitro. (**a**,**b**) Co-IP and proximity ligation assay (PLA) results showing that TRIM27 binding to FOXO1 was increased in HRGECs stimulated with LN plasma. Data were obtained from three independent experimental/culture repeats using the same pooled plasma preparations, with at least 10 randomly selected fields and 5 cells per field analyzed for each condition (Student’s *t*-test; scale bars: 10 μm). Red, PLA-positive signals; blue, nuclei. (**c**) Western blot results showing that forkhead box protein O1 (FOXO1) expression was decreased in HRGECs treated with LN plasma and appeared to be restored after MG132 treatment. Data are presented as the mean ± SD; one-way ANOVA with Tukey’s post hoc test. (**d**) Co-IP results showing that FOXO1 ubiquitination was increased in HRGECs treated with LN plasma. (**e**) FOXO1 stability was increased in HRGECs transfected with non-phosphorylated MT-TRIM27^S493A^. Protein levels were normalized to GAPDH and expressed as the percentage of remaining protein at time 0. Data are the mean ± SD from three independent experimental/culture repeats using the same pooled plasma preparations. Two-way ANOVA revealed a significant interaction (F = 15.52, *p* < 0.001), indicating a differential degradation rate between WT and mutant Ser493A. (**f**) Co-IP results showing that FOXO1 ubiquitination was suppressed in the MT-TRIM27^S493A^ group and appeared to be restored in the MT-TRIM27^S493D^ group. For (**c**), *n* = 3. For (**e**), *n* = 3. Experiments/cultures were repeated three times independently. *** *p* < 0.001 vs. control group; ### *p* < 0.001 vs. LN + DM group. Each point represents an individual sample.

Our previous research revealed that TRIM27 could regulate FOXO1 expression in HRGECs [13]. We detected the interaction between TRIM27 and FOXO1 using co-IP and PLA, finding that it was increased in HRGECs treated with LN plasma (Figure 7a,b). Growing evidence indicates that TRIM27 mediates the ubiquitination of various proteins; however, whether cytoplasmic TRIM27 contributes to FOXO1 ubiquitination remains unknown. FOXO1 expression decreased in HRGECs stimulated with LN plasma but increased after MG132 treatment (Figure 7c). FOXO1 ubiquitination was increased in the LN group compared with the control group (Figure 7d). In addition, the stability of FOXO1 was increased in the MT-TRIM27^S493A^ group compared with the WT-TRIM27 group (Figure 7e). FOXO1 ubiquitination was suppressed in the MT-TRIM27^S493A^ group but appeared to be restored in the MT-TRIM27^S493D^ group (Figure 7f). In summary, phosphorylation of TRIM27 at Ser^493^ may promote its cytoplasmic accumulation and may contribute to FOXO1 ubiquitination and HRGEC injury in LN.

## 4. Discussion

As a component of the glomerular filtration barrier, GEC integrity is crucial for kidney function [17,18,19]. GEC injury participates in multiple kidney diseases, including acute kidney injury and diabetic kidney disease [20,21,22]. The interaction between the endothelial glycocalyx and memory T cells accelerates LN progression [23]. Our previous research demonstrated GEC dysfunction in LN, including glycocalyx shedding and increased cell permeability [6].

TRIM27 participates in multiple biological processes [24,25,26,27]. Its localization can vary among different cells and tissues, which is closely related to its function. Although TRIM27 is reportedly overexpressed in renal cell carcinoma, our control samples showed no detectable levels. Thus, tumor contamination seems unlikely to explain our results—although we cannot exclude other more subtle alterations in these tissues that are independent of LN. Cytoplasmic TRIM27 in GECs was elevated in untreated LN patients, MRL/lpr mice, and cultured HRGECs, and this increase was moderately correlated with proteinuria. Given that proteinuria is influenced by multiple factors—including inflammation and podocyte dysfunction—this correlation should not be taken as evidence of a direct or exclusive role for TRIM27. However, additional experiments are required for validation because of the limited sample size and issues with tissue sampling. Inhibition of TRIM27 translocation by LMB reduced GEC damage in LN. Jung et al. found that cytoplasmic TRIM27 could bind to WNK1, regulating endosomal actin polymerization [28]. In addition, cytoplasmic TRIM27 mediated ULK1 polyubiquitination, regulating tumorigenesis [10]. We demonstrated that cytoplasmic TRIM27 may contribute to FOXO1 polyubiquitination, decreasing its abundance. However, our co-IP, MG132, and S493 mutant data are consistent with TRIM27-mediated regulation of FOXO1 ubiquitination, but FOXO1 is not established as a direct substrate of TRIM27. To establish this firmly, an E3-dead mutant, in vitro ubiquitination with purified proteins, or equivalent evidence is needed. FOXO1 knockdown and rescue would also help confirm that the endothelial phenotype is specifically mediated through FOXO1, especially because AKT can regulate FOXO1 on its own. We therefore view our findings as suggestive of a regulatory relationship, not as proof of direct enzyme–substrate interaction.

Nuclear TRIM27 plays a different function, such as regulating gene transcription. Zhao et al. reported that TRIM27 binds to the promoters of TFEB and CREB1, promoting transcription and inducing the expression of autophagy-related genes [11]. We observed increased nuclear TRIM27 expression in GECs in LN. However, the function and mechanism underlying this observation are unclear, requiring additional investigation.

We further explored the mechanism of TRIM27 translocation in GECs. LMB suppressed the cytoplasmic redistribution of TRIM27; LMB has been reported to inhibit nuclear transport by directly binding to CRM1 [14,29], an essential receptor that plays a role in cellular homeostasis by transporting substances from the nucleus to the cytoplasm [30,31,32]. Liu et al. found that CLOCK could bind to CRM1 and be translocated from the nucleus to the cytoplasm, accelerating hepatocellular carcinoma cell proliferation [33]. Additionally, BATF2 binds to CRM1 and is transferred out of the nucleus, promoting the growth of colorectal cancer [34]. In this study, the interaction between TRIM27 and CRM1 was verified by co-IP and PLA. Additionally, a CRM1 inhibitor suppressed TRIM27 cytoplasmic accumulation, indicating its important role in TRIM27 transport. However, the use of pharmacological CRM1 inhibitors does not exclude the possibility that other cytoplasmic redistribution cargo contributes to the observed endothelial effects. Although PLA and co-IP support a close association between TRIM27 and CRM1, they do not establish direct physical interaction. Future studies employing CRM1 genetic depletion, rescue experiments, in vitro binding assays, and mapping of the cytoplasmic redistribution signal in TRIM27 will be needed to confirm a direct and specific functional relationship.

Protein post-translational modifications are closely related to protein localization and function, including ubiquitination, methylation, acetylation, glycosylation, and phosphorylation [35,36,37]. Chiusa et al. reported that FUS phosphorylation induced its nuclear translocation, inducing transcription of collagen IV and participating in fibrosis [38]. Wei et al. found that TRIM24 phosphorylation mediated its transport from the nucleus to the cytoplasm, facilitating polyubiquitylation of EDC4 and AGO2, consequently leading to lipid accumulation in the liver [39]. Our study revealed that putative TRIM27 phosphorylation increased in HRGECs treated with LN plasma. Additionally, transfection with non-phosphorylated MT-TRIM27^S493A^ suppressed the interaction between TRIM27 and CRM1, reducing TRIM27 cytoplasmic accumulation and inhibiting HRGEC dysfunction. Transfecting the phospho-mimetic MT-TRIM27^S493D^ promoted the interaction between TRIM27 and CRM1, facilitating its cytoplasmic transport, which may contribute to injury of HRGEC. We note that these findings are based on a limited set of endpoints with modest replication and thus should be interpreted as preliminary. The function of TRIM27 Ser^493^ phosphorylation in mice needs further exploration, which may provide more evidence for it being a therapeutic target. In addition, while our Ser493A and Ser493D mutants point to a critical involvement of this residue in TRIM27 protein function, definitive evidence of actual endogenous phosphorylation—whether by LC–MS/MS or a site-specific antibody—will have to be addressed in follow-up work.

AKT, also known as protein kinase B, is a threonine/serine kinase that can be activated by numerous molecules and growth signals [40,41]. Activated AKT modulates various downstream proteins and participates in various biological processes, including regulating the cell cycle, promoting cell growth, inhibiting apoptosis, and mediating metabolism and immune responses [42,43,44,45]. Yang et al. showed that TRIM15 expression was increased in hepatocellular carcinoma when PI3K/AKT inhibitors were used in Huh7 and Hep3B cells [46]. AKT also promoted TRIM24 phosphorylation, leading to its accumulation in the cytoplasm [39].

Our prior research showed that activated AKT regulated TRIM27 expression [13]. In this study, an AKT inhibitor and activator suppressed and promoted TRIM27 phosphorylation, respectively. Simultaneously activating AKT and inhibiting CRM1 repressed TRIM27 cytoplasmic translocation, indicating a role for CRM1 in this process. We acknowledge that LN plasma is a complex mixture of inflammatory mediators, including cytokines, immune complexes, and complement components, which may activate multiple signaling pathways beyond PI3K/AKT. While our data support a role for AKT in TRIM27 regulation, we cannot exclude the possibility that other pathways contribute to TRIM27 expression and translocation. In addition, the inhibitor employed here, LY294002, acts as a broad PI3K pathway blocker rather than an AKT-specific agent. Nevertheless, we have not conducted in vitro kinase assays or AKT genetic deletion experiments to establish direct targeting. Thus, our findings suggest PI3K dependence and a potential role for AKT, although additional studies are required to validate whether AKT directly phosphorylates TRIM27 at Ser^493^, both in vivo and in vitro.

In addition, we observed a positive correlation between TRIM27 staining and proteinuria in LN patients. However, our sample size (n = 30) and the heterogeneous class distribution precluded reliable multivariate adjustment. We therefore regard this as an exploratory finding requiring validation in larger cohorts. Nevertheless, this clinical association, combined with our functional data, supports a potential involvement of TRIM27 in LN pathogenesis.

The MRL/lpr model used here is characterized by systemic autoimmunity and multi-organ inflammation, not isolated renal disease. TRIM27 redistribution in glomerular endothelial cells could therefore be influenced by the generalized inflammatory milieu, not only by kidney-specific signals. Although our LN plasma experiments partially replicate the local environment, circulating cytokines or immune complexes might still contribute. We acknowledge this limitation in our interpretation and suggest future studies using conditional models to dissect renal versus systemic contributions.

## 5. Conclusions

Taken together, our data indicate that PI3K signaling, potentially involving AKT, may promote putative TRIM27 phosphorylation and its interaction with CRM1, thereby facilitating cytoplasmic accumulation and subsequent changes in FOXO1 ubiquitination and stability, which may contribute to endothelial injury.

## Data Availability

The original contributions presented in this study are included in the article/Supplementary Material. Further inquiries can be directed to the corresponding authors.

## Supplementary Materials

The following supporting information can be downloaded at https://www.mdpi.com/article/10.3390/cells15181696/s1. Figure S1. Cell viability of HRGECs treated with LMB, selinexor, LY294002, SC79, or MG132 under the same conditions used for functional assays. Figure S2. Shown are the original western blot images corresponding to Figure 1 in the main text. Figure S3. Shown are the original western blot images corresponding to Figure 2 in the main text. Figure S4. Shown are the original western blot images corresponding to Figure 3 in the main text. Figure S5. Shown are the original western blot images corresponding to Figure 4 in the main text. Figure S6. Shown are the original western blot images corresponding to Figure 6 in the main text. Figure S7. Shown are the original western blot images corresponding to Figure 7 in the main text. Table S1: Information on Lupus Nephritis Patients Using Renal Biopsy Samples [9]. Table S2. Data from lupus nephritis patients using lupus nephritis plasma.

## Abbreviations

The following abbreviations are used in this manuscript:

TRIM27	Tripartite motif-containing 27
GEC	Glomerular endothelial cell
LN	Lupus nephritis
CRM1	Chromosome region maintenance 1
AKT	Protein kinase B
FOXO1	Forkhead box protein O1
HRGEC	Human renal glomerular endothelial cell
IF	Immunofluorescence
LMB	Leptomycin B
ET	Absolute ethanol
VCAM-1	Vascular cell adhesion molecule-1
ELISA	Enzyme-linked immunosorbent assay
FITC-BSA	Fluorescein isothiocyanate–bovine serum albumin
Co-IP	Co-immunoprecipitation
PLA	Proximity ligation assay
DMSO	Dimethyl sulfoxide
MG132	Carbobenzoxy-L-leucyl-L-leucyl-L-leucinal
CHX	Cycloheximide
IHC	Immunohistochemistry
NO	Nitric oxide
ZO-1	Zonula occludens-1
