# Supplementary material for: CRM1-Dependent Cytoplasmic Accumulation of TRIM27 Is Associated with Glomerular Endothelial Cell Injury in Lupus Nephritis
Source: Cells. 2026 Sep 18;15(18):1696. doi: 10.3390/cells15181696 (PMC13604956; doi:10.3390/cells15181696)
Supplement: Supplementary file 1 [file cells-15-01696-s001.zip › Figure S1.pdf]

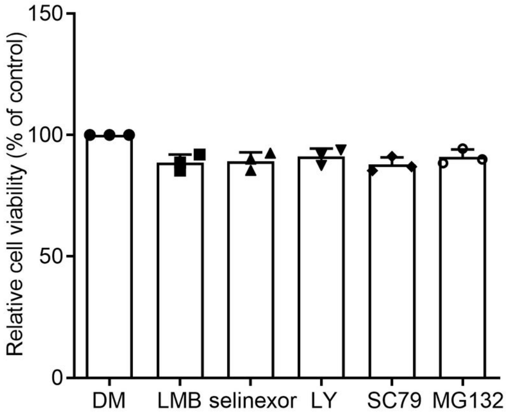

Figure S1. Cell viability of HRGECs treated with LMB, selinexor, LY294002, SC79, or MG132 under the same conditions used for functional assays. CCK-8 readings were normalized to vehicle controls (100%). Data are presented as the mean ± SD from three independent experiments, each performed in triplicate technical replicates. No statistically significant differences were observed between any treatment group and the vehicle control (one-way ANOVA with Tukey's post hoc test; all $P > 0.05$).
