# Supplementary material for: CRM1-Dependent Cytoplasmic Accumulation of TRIM27 Is Associated with Glomerular Endothelial Cell Injury in Lupus Nephritis
Source: Cells. 2026 Sep 18;15(18):1696. doi: 10.3390/cells15181696 (PMC13604956; doi:10.3390/cells15181696)
Supplement: Supplementary file 1 [file cells-15-01696-s001.zip › Figures S2-S7/Figures S2-S7. Original Images for Blots.pdf]

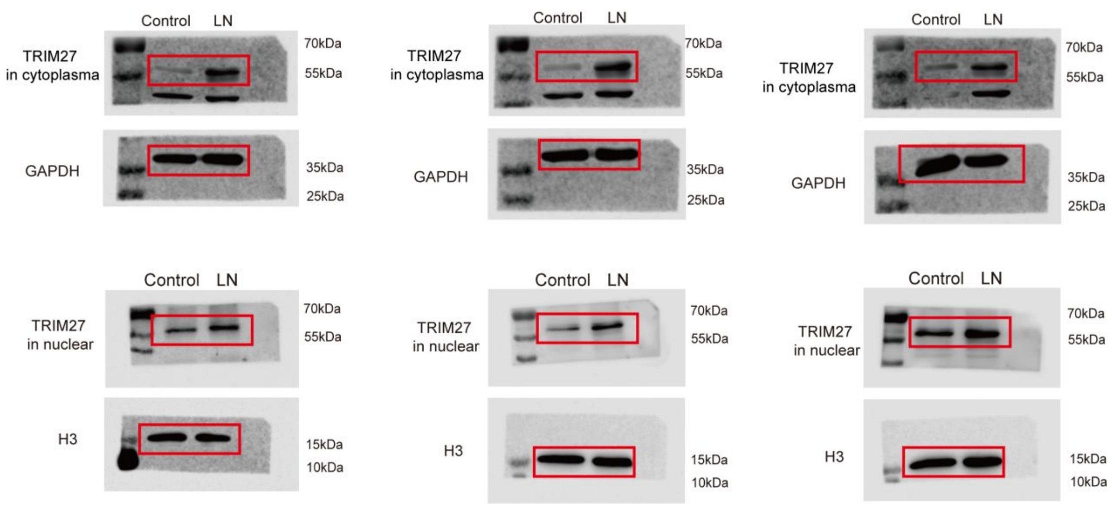

Figure S2. Shown are the original western blot images corresponding to Figure 1 in the main text. The membrane was cut with a scalpel to enable simultaneous detection of multiple proteins on a single blot. The quantified bands are indicated by solid red boxes.

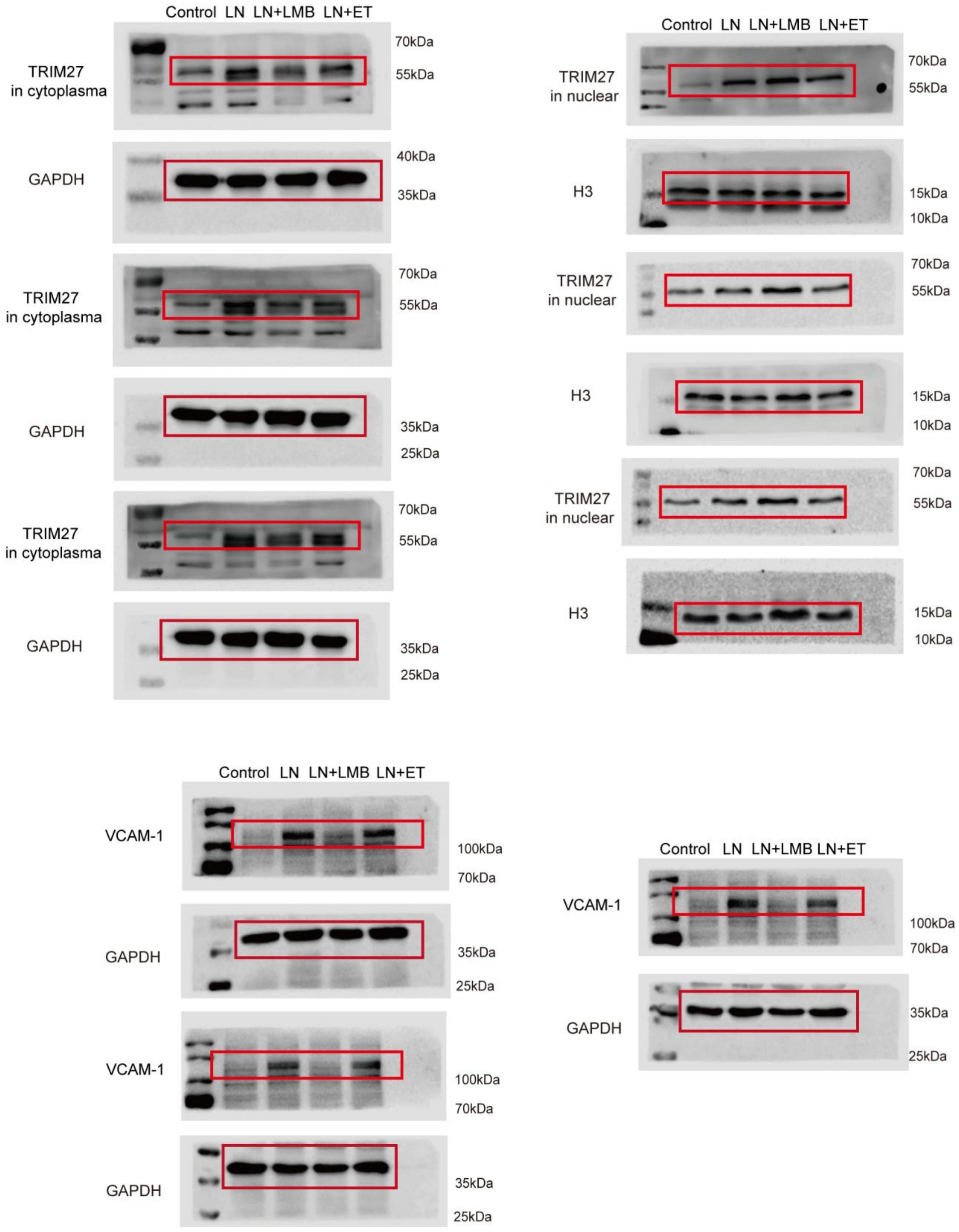

Figure S3. Shown are the original western blot images corresponding to Figure 2 in the main text. The membrane was cut with a scalpel to enable simultaneous detection of multiple proteins on a single blot. The quantified bands are indicated by solid red boxes.

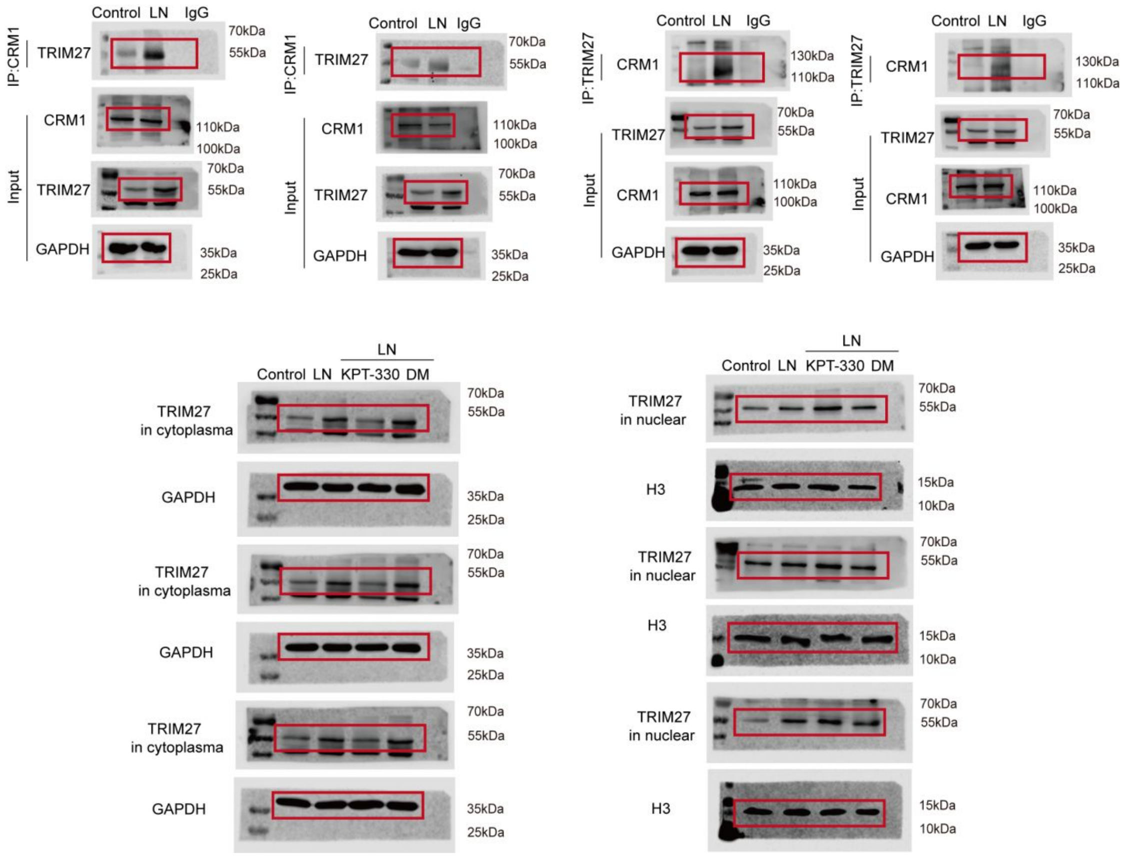

Figure S4. Shown are the original western blot images corresponding to Figure 3 in the main text. The membrane was cut with a scalpel to enable simultaneous detection of multiple proteins on a single blot. The quantified bands are indicated by solid red boxes.

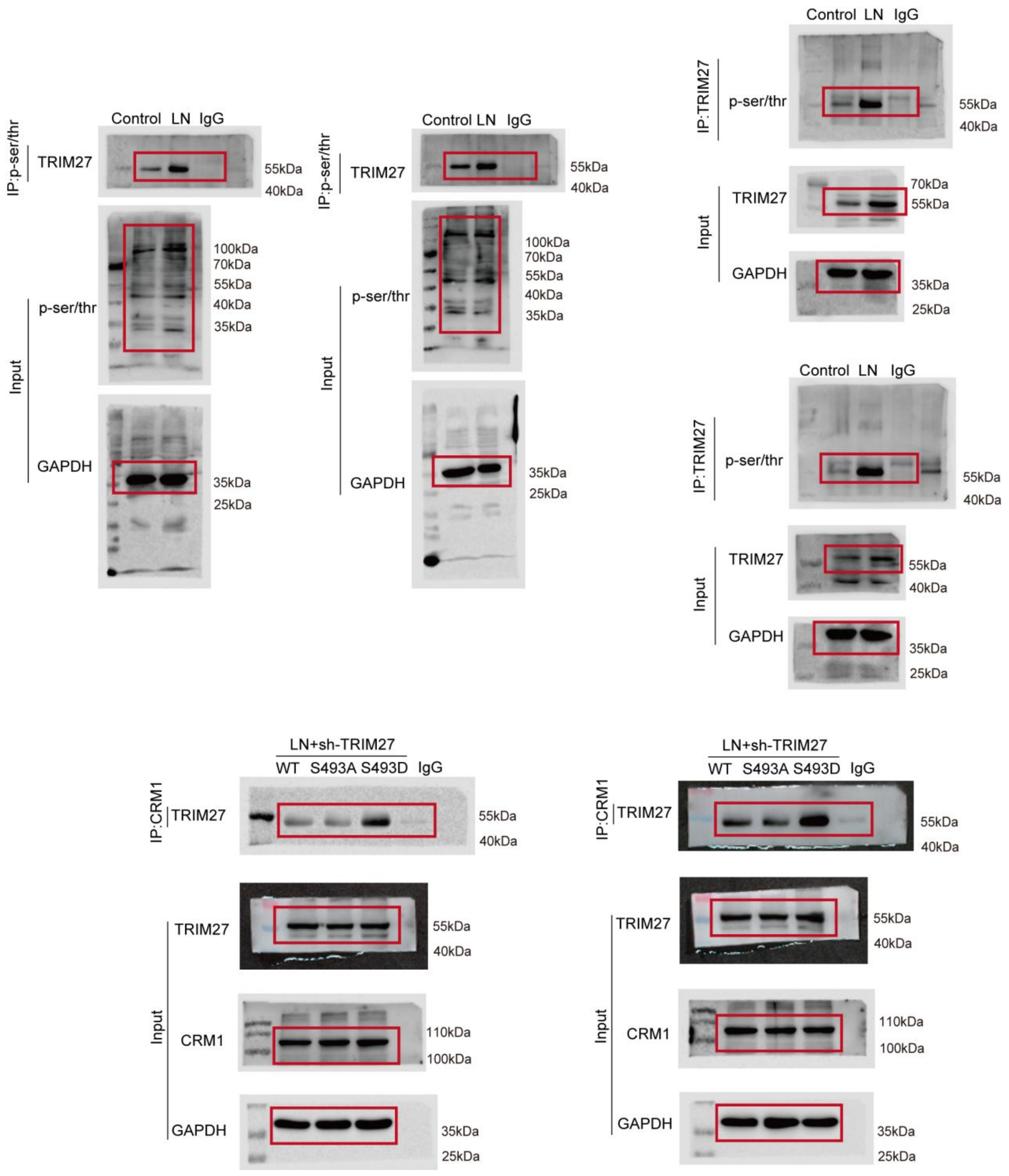

Figure S5. Shown are the original western blot images corresponding to Figure 4 in the main text. The membrane was cut with a scalpel to enable simultaneous detection of multiple proteins on a single blot. The quantified bands are indicated by solid red boxes.

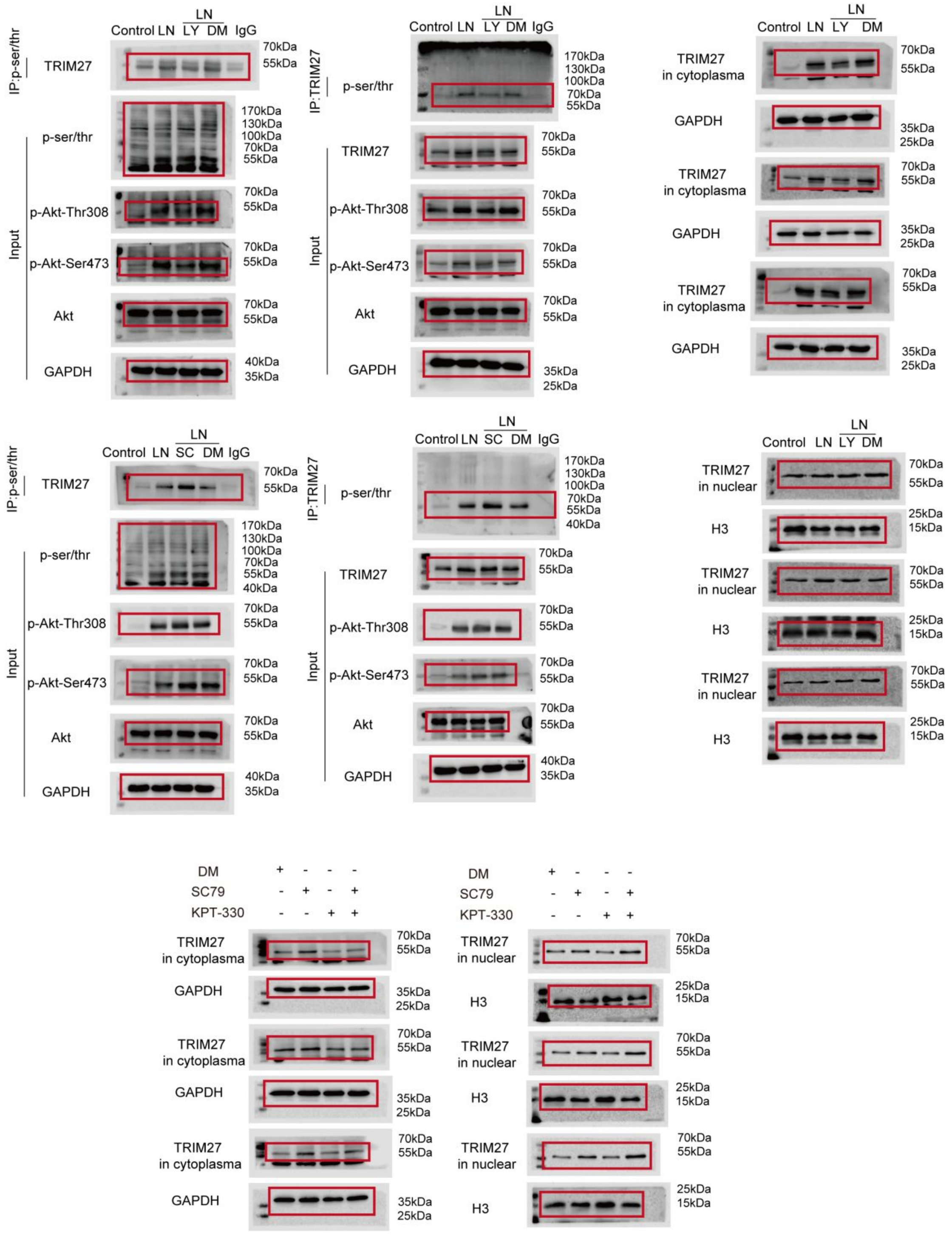

Figure S6. Shown are the original western blot images corresponding to Figure 6 in the main text. The membrane was cut with a scalpel to enable simultaneous detection of multiple proteins on a single blot. The quantified bands are indicated by solid red boxes.

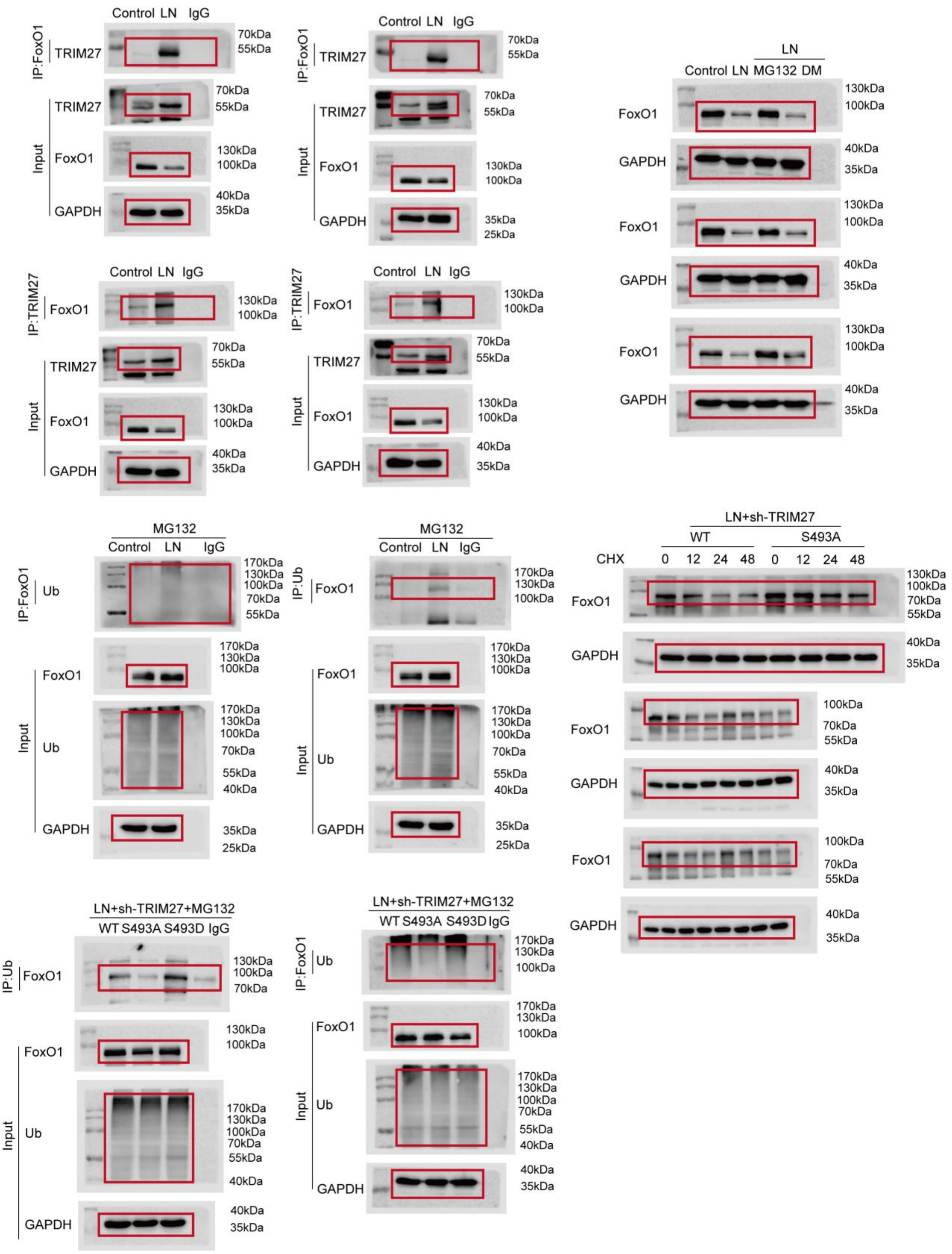

Figure S7. Shown are the original western blot images corresponding to Figure 7 in the main text. The membrane was cut with a scalpel to enable simultaneous detection of multiple proteins on a single blot. The quantified bands are indicated by solid red boxes.
